# Evaluation of kidney injury and metabolomic analysis in adulthood in a non-obese hyperglycemic female mouse model after birth with low birthweight

**DOI:** 10.1186/s12882-025-04290-1

**Published:** 2025-07-02

**Authors:** Shoichi Shimizu, Nobuhiko Nagano, Daichi Katayama, Kengo Matsuda, Wataru Tokunaga, Kimitaka Nakazaki, Ryoji Aoki, Kazumasa Fuwa, Ichiro Morioka

**Affiliations:** Department of Pediatrics and Child Health, Nihon University School of Medicine, Tokyo, Japan

**Keywords:** Chronic kidney disease (CKD), Non-obese-hyperglycemia, Uterine artery ischemia, Metabolomic analysis, Developmental origins of health and disease (DOHaD), Small-for-gestational-age (SGA)

## Abstract

**Background:**

Low birthweight infants have high risk of developing chronic kidney disease (CKD) in later in life, however, the pathogenesis of this disease remains unclear. This study aimed to investigate the underlying mechanism using a low birthweight-non-obese hyperglycemic adulthood mouse model.

**Methods:**

Pregnant ICR-strain mice underwent uterine artery ligation at day 16.5 of gestation to induce fetal hypoxia (ischemic group, I). Female newborns were weaned at 4 weeks of age and fed a normal diet until 8 weeks of age (*n* = 10). The group I was compared to the control group (C) regarding the body weight, tubular injury markers, renal function, pathology, and metabolome analysis.

**Results:**

Group I were born with a low birth weight (group I: C = 1.4:1.9 g, *p* < 0.01), which persisted after birth. By 8 weeks of age, there were minimal changes in kidney histopathology between the two groups. However, group I showed an increase in markers for detection of CKD, such as urinary β2-microglobulin levels (group I: C = 116:26 µg/L), albumin levels (group I: C=0.14:0.07 mg/gCr) (both *p* < 0.01) and serum creatinine levels (group I: C= 0.18:0.12 mg/dL, *p* < 0.05). Furthermore, kidney metabolomic analysis revealed notable differences between the two groups, particularly in succinic acid, S-adenosylmethionine, and N1-methyl-4-pyridone-5-carboxamide (4PY), which are closely linked to kidney injury.

**Conclusion:**

The low birthweight-non-obese hyperglycemic mouse model may develop CKD in adulthood, potentially caused by increased renin activity related to succinic acid and tissue injury related to S-adenosylmethionine and 4PY.

**Clinical trial number:**

Not applicable.

**Supplementary Information:**

The online version contains supplementary material available at 10.1186/s12882-025-04290-1.

## Introduction

In Japan, although the birthrate is declining, the proportion of low birth weight infants has not decreased. Low birth weight infants, especially small-for-gestational-age (SGA) infants, are prone to developing chronic kidney disease (CKD) in the future [1], with several mechanisms known to cause this. Among these mechanisms, a decreased number of nephrons at birth leads to hyperfiltration of the residual glomeruli. Subsequently, elevated intraglomerular pressure can cause focal glomerulosclerosis, which results in hypertension, and ultimately, kidney injury, creating a negative loop [2]. It has also been hypothesized that the pathological mechanism of CKD involves metabolic abnormalities in the inner mitochondrial membrane via a breakdown in redox reactions, which leads to mitochondrial injury. Moreover, an increase in the adenosine monophosphate (AMP)/adenosine triphosphate ratio in the kidney causes intracellular energy insufficiency in AMP-activated protein kinase [3].

In experiments using fetal malnourished rats, we proposed that epigenetic abnormalities in stem cells in the kidney may lead to kidney injury and hypertension in adulthood [4]. However, this fetal malnutrition model may cause changes in the metabolic state and body environment of pregnant mothers due to an extremely low-protein diet, and it remains unclear whether this model can be applied to low birthweight infants in humans. Therefore, we successfully generated an SGA mouse model using intrauterine ischemia manipulation. We reported that this model develops hyperglycemia even after birth, despite being low weight, i.e., non-obese, and becoming an adult (a non-obese hyperglycemic mouse model that develops after birth with low birthweight, low birthweight-non-obese hyperglycemic mouse model) [5]. We sought to analyze these low birthweight-non-obese hyperglycemic model mice given that this model is thought to be closer to the actual clinical situation of low birthweight infants in humans.

The purpose of this study was to clarify the presence of CKD in this mouse model by assessing kidney histomorphology and kidney function using urinalysis, blood analysis and pathological examination. We also performed metabolomic analysis of kidney tissue to elucidate the pathogenesis of kidney injury. The results may help to elucidate the mechanisms involved in CKD in low birthweight infants in adulthood.

## Materials and methods

### Creation of a low birthweight, non-obese, hyperglycemic mouse model

This study was conducted in accordance with the ARRIVE guidelines, and the protocol was approved by the Nihon University Animal Care and Use Committee (protocol number: AP20MED003-1 [April 3, 2020]). ICR-strain mice (Sankyo Lab Service Co., Ltd. Tokyo, Japan) were obtained on gestational day 12. All mice were fed a normal diet (moisture: 7.9%, crude fat: 5.1%, crude protein: 23.1%, crude ash: 5.8%, crude fiber: 2.8%, and soluble solids: 55.3%; Oriental Yeast Co., Ltd., Tokyo, Japan) and had free access to water. Female mice were given the same diet and water until weaning after birth. On day 16.5 of pregnancy, the lower abdomen was incised under isoflurane inhalation anesthesia (induction 5%, maintenance 2%). The animals were fully anesthetized and unconscious. In the ischemia group (group I), the mother mice were preheated to 37.5 °C on a hot plate. The uterine and ovarian arteries were exposed, and blood flow was blocked by clipping a total of four sites: the left and right uterine arteries, and the ovarian arteries distal to the ovaries on both sides. with a clip for 15 min to induce fetal hypoxia and malnutrition [6]. The uterine artery clip was removed, the fetus was returned to the mother’s abdomen, and the abdomen was sutured. The control group (group C) underwent a lower abdominal incision under similar anesthesia.

The newborns were kept under the care of their mothers. Female newborns in both groups were weaned at 4 weeks of age and fed a normal diet until 8 weeks of age (Online Resource 1). The pups were weighed at birth and twice a week thereafter until they reached 8 weeks of age, at which point the weight gain plateaued. Mice aged 8 weeks are considered equivalent to adult humans [7].

### Confirmation of the kidney tissue injury phenotype

Female newborns in groups I and C were weaned at 4 weeks of age and fed a normal diet until 8 weeks of age (*n* = 10 per group). The mice were weighed at birth and weekly thereafter until 8 weeks of age. At 7–8 weeks of age, the urinary β2-microglobulin and albumin levels were measured. For the measurements, 24-h urine samples were collected from mice housed in metabolic cages for experimental animals. The samples were stored at − 20 °C, and urinary β2-microglobulin was measured by latex agglutination and albumin by immunoturbidimetry (SRL, Inc., Tokyo, Japan). Finally 8 weeks of age, the rats were euthanized under deep anesthesia using isoflurane (5%), Blood was then drawn from the heart, and the kidneys were removed.

Blood was centrifuged at 3000 rpm for 5 min at room temperature, and serum was stored at − 20 °C. Serum creatinine levels were measured using a Dri-Chem slide (Fujifilm Corporation, Tokyo, Japan).

The kidneys were fixed in 10% formalin followed by 70% alcohol replacement, and kidney sections were prepared. Periodic acid-Schiff staining was performed to assess the histological features. The kidney sections were examined under a light microscope at 100× magnification to evaluate the renal cortex. Glomeruli were counted in 10 fields per section, and the total number was recorded. The glomerular length diameter was measured in a total of 107 glomeruli in group I and 127 glomeruli in group C, combining data from all individuals. Only glomeruli in which both the vascular pole and urinary pole were clearly identifiable were included, ensuring near-equatorial sections. The glomerular injury score (GIS) and tubulointerstitial injury score (TIS) were calculated based on the method outlined by Raij et al. [8] to determine the proportion of sclerotic glomeruli.

### Metabolomic analysis

Metabolomic analysis was performed to investigate kidney function and glucose metabolism. The metabolites were extracted as follows: Approximately 50 mg of frozen kidney tissue was removed from female mice (8 weeks old, *n* = 3 per group) and placed in homogenization tubes with zirconia beads (5 mmφ and 3 mmφ); next, an internal standard (H3304-1002, Human Metabolome Technologies, Inc. (HMT), Tsuruoka, Yamagata, Japan) was added, and the tissue was homogenized for 120 s at 4 °C for two cycles at 1500 rpm using a bead shaker (Shake Master NEO, Bio Medical Science, Tokyo, Japan); the homogenate was centrifuged at 2300 x g for 5 min at 4 °C, and the supernatant (400 µL) was centrifuged at 9100 × g for 120 min at 4 °C using a Millipore 5-kDa cutoff filter (Human Metabolome Technologies, Inc.) to remove the polymers; and finally, the filtrate was dried under vacuum and redissolved in 50 µL Milli-Q water for metabolomic analysis. Metabolomic analysis was performed using capillary electrophoresis time-of-flight mass spectrometry as previously reported [9, 10] using an Agilent CE capillary electrophoresis system (Agilent Technologies, Inc., Santa Clara, CA, USA). The spectrometer was scanned from 50 to 1000 m/z, and peaks were extracted using integrated software (Keio University, Tsuruoka, Yamagata, Japan) to obtain data on m/z, migration time, and peak area [11]. Peaks were determined using metabolite databases based on m/z values and migration times. Peak areas were standardized using internal standards and sample volumes to obtain relative metabolite levels. Principal component analysis and hierarchical cluster analysis were performed as previously reported [12]. The detected metabolites were plotted on a metabolic pathway map, as previously reported [13].

### Statistical analysis

Statistical analysis was performed using JMP ver. 14. The Wilcoxon/Kruskal–Wallis test and Welch’s t-test were calculated, where statistical significance was set to p-value < 0.05.

## Results

### Birth weight and weight change up to 8 weeks of age

**Fig. 1:**
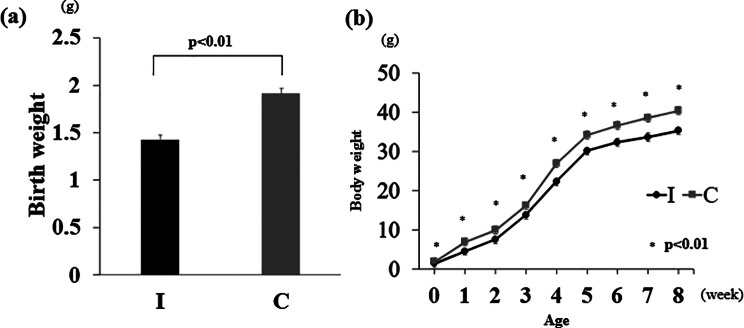
Birth weight and changes in body weight gain. (**a**) The birth weight was measured on the first postnatal day(*n* = 10 per group). (**b**) Changes in weight gain from birth to 8 weeks of age(*n* = 10 per group). Ischemia group: I, Control group: C. Data are presented as the mean ± standard error of the mean

The mean birth weight was 1.5 ± 0.05 g (mean ± standard error: SE) in group I and 1.8 ± 0.05 g (mean ± SE) in group C, and low birthweight pups were born due to intrauterine ischemia (*p* < 0.01) (Fig. 1a). Group I had consistently underweight than group C thereafter, until at 8 weeks of age. (*p* < 0.01) (Fig. 1b).

### Urinary β2-microglobulin and albumin levels at 7 weeks of age

**Fig. 2:**
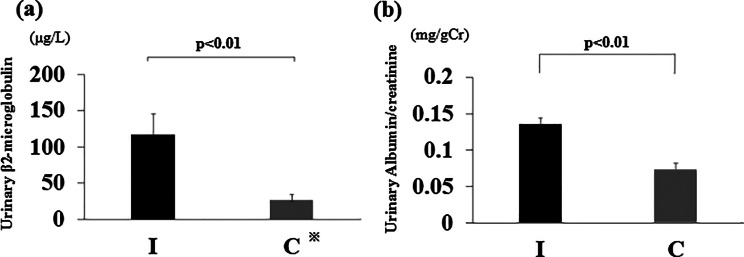
Markers of urinary tubular dysfunction. (**a**) Urinary β2-microglobulin at 7 weeks of age. ※Group C contained three samples with sensitivities of 10 µg/L or less. (**b**) Urinary albumin/creatinine ratio at 7 weeks of age. Ischemia group: I, Control group: C. Data are presented as the mean ± standard error of the mean (*n* = 6 per group)

The mean urinary β2-microglobulin (Fig. 2a) and albumin (Fig. 2b) levels at 7 weeks of age were significantly higher in group I than in group C (both *p* < 0.01).

### Serum creatinine level at 8 weeks of age

**Fig. 3:**
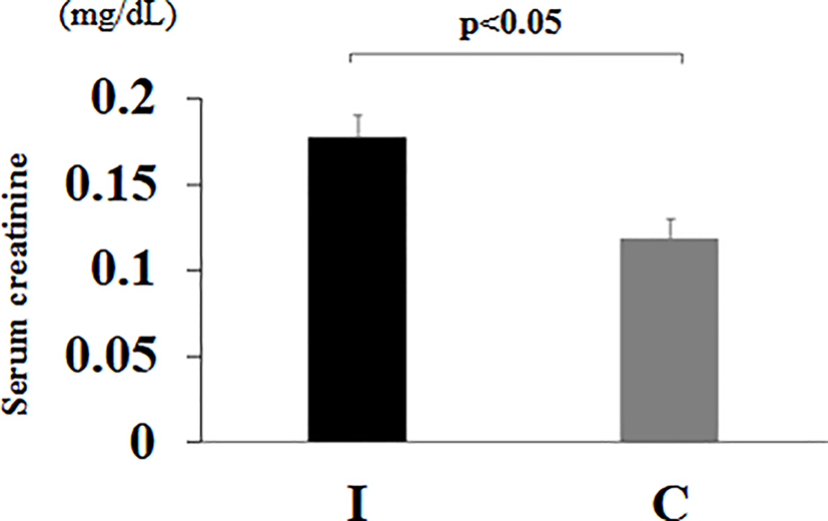
Serum creatinine level at 8 weeks of age. Ischemia group: I, Control group: C. Data are presented as the mean ± standard error of the mean (*n* = 6 per group)

The mean serum creatinine level at 8 weeks was significantly higher in group I than in group C (*p* < 0.05) (Fig. 3).

### Kidney pathology at 8 weeks of age

**Fig. 4:**
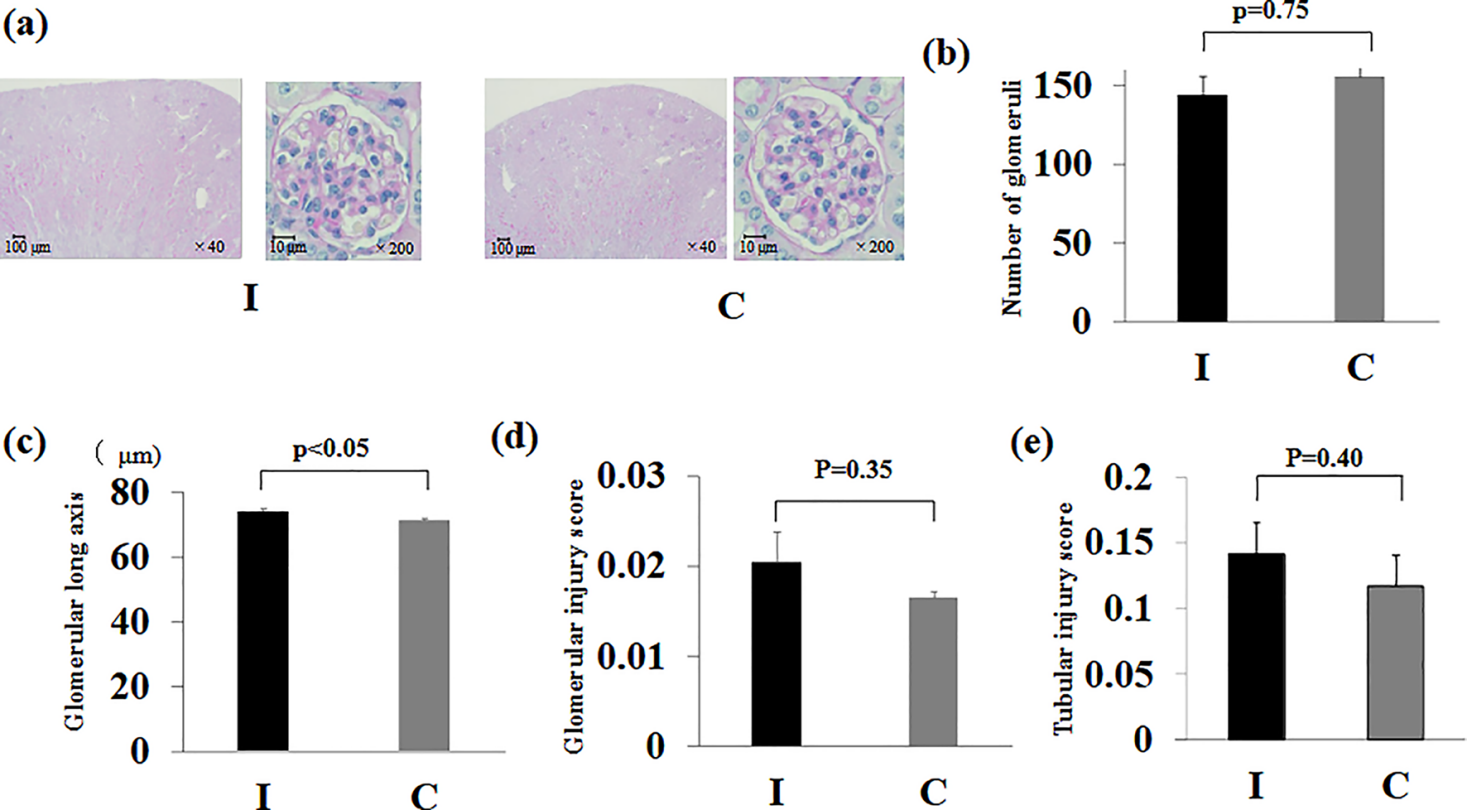
Pathological findings of the kidney. (**a**) Pathological image. Periodic acid-Schiff stain (×40, ×200). (**b**) Number of glomeruli. (**c**) Glomerular long axis. (**d**) Glomerular injury score. (**e**) Tubular injury score. Ischemia group: I, Control group: C. Data are presented as the mean ± standard error of the mean (*n* = 6 per group)

The kidney pathology findings at 8 weeks of age were compared between groups, revealing no significant differences in glomerular counts, GIS and TIS (*p* = 0.75, *p* = 0.35, *p* = 0.40). The glomerular length diameter was significantly longer in group I than in group C (*p* < 0.05) (Fig. 4).

### Metabolome analysis

**Fig. 5:**
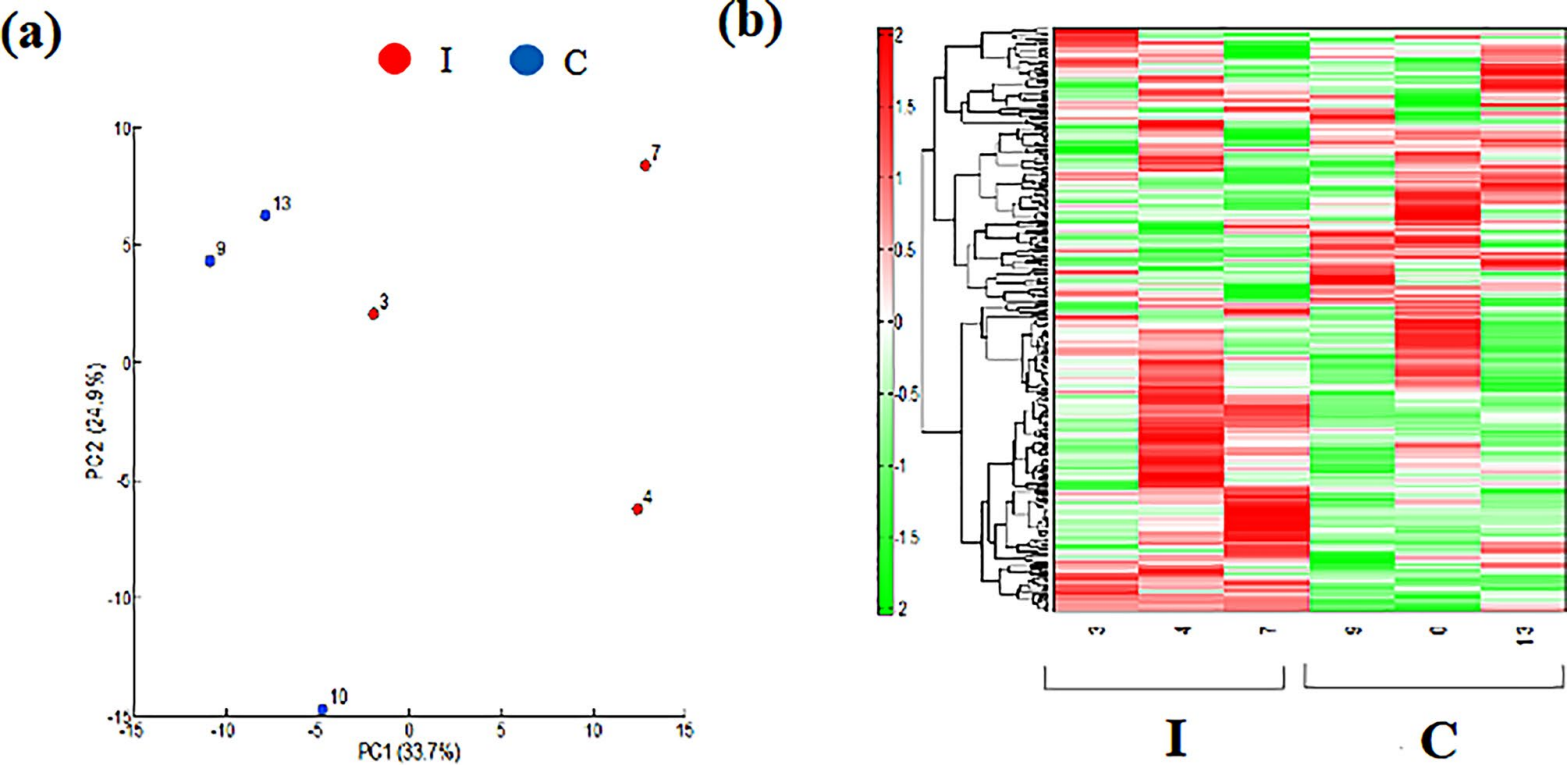
Metabolite analyses of kidney tissue. (**a**) Principal component (PC) analysis. (**b**) Heatmap display of the hierarchical cluster analysis. Ischemia group: I, Control group: C. *n* = 3 per group

Metabolomic analysis of the kidney revealed a clear difference between groups I and C in the heatmap display of principal component analysis and hierarchical cluster analysis(Fig. 5). Significant differences between the two groups were observed for the compounds outlined in Table 1. Furthermore, when an investigation was performed based on the Human Metabolome Database, the compounds listed in Table 2 were classified as renal disease and uremic toxins; of these, the two groups showed a N1-methyl-4-pyridone-5-carboxamide (4PY) was significantly higher in group I than in group C. The raw data is provided in the supplementary file. Further information that supports the findings of the metabolome analysis are available from HMT (Tsuruoka, Yamagata, Japan), but restrictions apply to the availability of these data, which were used under license for the current study, and so are not publicly available. Data are however available from the authors upon reasonable request and with permission of HMT (Tsuruoka, Yamagata, Japan).

**Table 1:** Compounds for which a significant difference was observed between the two groups

Compound name	Comparative analysis	
	Group I^a^ vs. C^b^	
	Ratio	*p*-value
Argininosuccinic acid	1.5	0.021
Ascorbic acid	0.5	0.004
Cytidylic acid	0.7	0.032
Flavin adenine dinucleotide divalent	0.8	0.046
Hypotaurine	1.3	0.024
Inosine	0.8	0.034
Isovalerylcarnitine	2.4	0.012
*myo*-Inositol 1-phosphate *myo*-Inositol 3-phosphate	1.4	0.039
N1-Methyl-4-pyridone-5-carboxamide (4PY)	2.6	0.049
*N*^5^-Ethylglutamine	2.1	0.011
Ribulose 5-phosphate	0.7	0.008
*S*-Adenosylmethionine	0.5	0.002
*S*-Carboxymethylcysteine	1.6	0.029
Spermidine	1.5	0.024
Stachydrine	2	0.043
Succinic acid	1.8	0.005
Taurocholic acid	0.6	0.027

a: Ischemia group, b: Control group

**Table 2:** Examination based on the human metabolome database

Category	Compound name	Comparative analysis	
		Group I^a^ vs. C^b^	
		Ratio	*p*-value
Renal failure, kidney disease, uremic toxin	3-Indoxylsulfuric acid	2.3	0.231
Renal failure	Dimethylarginine	1.2	0.391
Renal failure, kidney stone	Citric acid	1.4	0.476
Kidney disease, renal function	Creatinine	1.7	0.127
Renal failure, aciduria, kidney stone	Glyceric acid	2.3	0.085
Kidney disease, uremic toxin	Guanidinosuccinic acid	1.4	0.255
Chronic renal failure	His	1	0.913
Kidney disease	Lys	1.2	0.344
Renal failure	*N*-Acetylneuraminic acid	1	0.76
Renal failure	*N*^1^-Methyl-4-pyridone-5-carboxamide (4PY)	2.6	0.049
Renal disease	Symmetric dimethylarginine	1.3	0.295
Kidney failure	Taurine	0.9	0.504
Chronic renal failure, hemodialysis	Taurocyamine	1.1	0.579
Kidney failure	Trimethylamine *N*-oxide	2.5	0.142

a: Ischemia group, b: Control group

## Discussion

A low birthweight-non-obese hyperglycemic mouse model was generated using intrauterine ischemia. The body weight of mice was monitored from birth, and blood and urine tests related to kidney function were performed in early adulthood. Metabolomic analysis and a histopathological search of kidney tissue were also performed.

In a previously reported fetal growth restriction rat model, the histological findings at 32 weeks of age showed severe glomerular sclerosis and increased glomerular diameter due to long-term kidney dysfunction [14]. In contrast, in this experiment, the low birthweight-non-obese hyperglycemic model mice showed only slight histopathological changes at the relatively young age of 8 weeks. The only significant difference observed was that the glomerular length diameter was greater in group I compared to group C. Subsequently, although the difference was not statistically significant, group I tended to have a lower glomerular count. This finding likely reflects compensatory glomerular hypertrophy resulting from reduced nephron endowment [15]. Furthermore, we consider that the increased glomerular diameter represents early glomerular hypertrophy in response to hyperfiltration, which may, in turn, contribute to the observed increase in albuminuria [2]. Although urinary β2-microglobulin was elevated in our model, no overt pathological alterations were observed in the renal tubules under light microscopy. This discrepancy may be attributed to the fact that urinary β2-microglobulin is a highly sensitive marker of proximal tubular dysfunction, which can precede detectable histological changes. Indeed, increased β2-microglobulin levels can occur in the absence of visible structural abnormalities on light microscopy. Previous studies have similarly reported that tubular dysfunction can precede morphological injury in various pathological states [16]. Therefore, the observed increase in urinary β2-microglobulin in our model may reflect early, subclinical tubular injury that is not yet apparent on conventional histopathological evaluation. Although overt pathological findings did not support a diagnosis of CKD, the presence of elevated tubular injury markers and albuminuria in the low birthweight-non-obese hyperglycemic mice suggests early signs of CKD. This tendency is consistent with findings in a previous fetal growth restriction rat model evaluated at a younger age [17].

We also examined compounds that showed significant differences between the two groups in metabolomic analysis of the kidneys. Succinic acid, S-adenosylmethionine, and 4PY, which are known to be associated with kidney disease, were examined. The succinic acid content was significantly higher in group I than in group C. Hyperglycemia causes high succinate production in mitochondria. Previous reports have shown that succinate activates prorenin in the distal tubules where succinate receptors are present, producing renin and contributing to the onset of kidney injury [18], suggesting that the increase in renin activity induced by succinate may be involved in the onset of CKD in low birthweight-non-obese hyperglycemic model mice. S-adenosylmethionine, which showed significantly lower values in group I than in group C, is a direct methyl group donor of methionine [19]. Therefore, it is suggested that in the low birthweight-non-obese hyperglycemic mouse model, a shortage of S-adenosylmethionine in the tissues led to a decrease in the rate of methylation, which may have led to a decrease in the tissue repair ability of the kidney younger age.

Physical property classification using the Human Metabolome Database revealed that 4PY, an indicator of kidney dysfunction and the final product of nicotinamide adenine dinucleotide (NAD), was significantly higher in group I than in group C.

Poly (ADP-ribose) polymerase (PARP) plays an important role in the degradation of NAD. PARP is a nuclear enzyme that is deeply involved in various physiologically important events such as gene expression regulation, cell differentiation, apoptosis, DNA replication, and DNA repair [20]. It has been reported that DNA damage increases PARP activity several-fold [21]. As a result, intracellular NAD is rapidly depleted, resulting in the accumulation of nicotinamide. Nicotinamide is converted back to NAD or metabolized to 4PY [22]. In other words, 4PY increases at sites of DNA damage and can serve as a marker of tissue injury, including kidney tissue injury. Considering these mechanisms, the results of this metabolomic analysis showing that 4PY in kidney tissue was significantly higher in group I than in group C suggest that DNA damage may have occurred in kidney tissue in the low birthweight-non-obese hyperglycemic model mice.

Furthermore, in a previous report using the same mouse model, we reported that serum NAD levels were significantly lower in group I than in group C (*p* = 0.010), which is in agreement with the 4PY movement in kidney tissue in this study [2]. In the same study, the reason for the change in NAD was proposed to be due to ischemia and reperfusion during the fetal period causing oxidative stress and a decrease in mitochondrial function [23–26]. This further reinforces the possibility that oxidative stress due to intrauterine ischemia in a low birthweight-non-obese hyperglycemic mouse model may cause mitochondrial dysfunction after birth and ultimately DNA damage in kidney tissue. Consequently, it is inferred that when the repair of DNA damage collapsed, CKD develops. We need to consider measuring NAD in the kidneys in future studies. These findings suggest that DNA damage in this model may affect both distal tubules, which express succinate receptors, and proximal tubules, which depend on mitochondrial function and are potentially impacted by 4PY [27]. However, in this study, metabolomic analysis was performed without separating tubules and glomeruli. This limitation should be addressed in future investigations.

There are several limitations to this study. First, this study used only female mice. The non-obese hyperglycemic mouse model used in this study was based on a previous study using females [5]. The rationale for this is that many young-onset non-obese type 2 diabetes patients in humans were female [28]. We limited the use of female mice in this study because the renal function of males and females is different. A recent report using a model of uterine arteriovenous narrowing similar to the present study found glomerular defects in male mice, but no significant changes in female mice [29]. The paper considers the following points, in general, men are at a higher risk of CKD progression with a more rapid decline than women, and low-birth-weight has a greater association with CKD progression in men. Mechanistically, female hormones, such as estrogen, have been considered renoprotective factors. Thus, with regards to the effect of sex on kidney damage, their low-birth-weight model also imitated the phenotype observed in the clinical setting. As our analysis was conducted exclusively in females, the relatively mild histological alterations observed are considered to be consistent with the findings reported in the aforementioned study.

Second, we did not discriminate between individual mice, and we were unable to perform statistical corrections for glomerular length diameter measurements. Therefore, it is possible that the accuracy of the statistical analysis was compromised.

Third, it was performed before actual histological changes were added due to the short rearing period. However, epigenetic changes acquired during the perinatal period and immediately after birth are a predisposition to cardiometabolic risk factors, including lifelong kidney disease [30]. Therefore, it is important to evaluate kidney tissue before the addition of histological changes. In this regard, we did not collect blood and urine samples at birth or at 4 weeks of age. Such early time-point analyses may have provided more detailed insights into the temporal progression of renal dysfunction and metabolic alterations. In addition, the inclusion of other tubular injury markers such as urinary N-acetyl-β-D-glucosaminidase could have further strengthened the evaluation of tubular pathology. We recognize these as limitations of the current study and plan to address them in future experiments.

Fourth, the kidneys used for metabolomic analysis were not perfused prior to dissection. Therefore, it is possible that the observed differences in metabolite profiles may, at least in part, reflect contributions from blood-derived metabolites.

Fifth, we did not create knockout models for each compound extracted via metabolomic analysis in this study; thus, we cannot prove that the effects of each compound are independent, and we cannot deny the possibility that the effects are due to multiple factors. These are issues to be considered in the future.

## Conclusion

In the low birthweight-non-obese hyperglycemic mouse model, kidney tubular injury and microalbuminuria were observed from early adulthood, and kidney function also changed. The possibility of CKD through long-term rearing was recognized, and the pathology was suggested to involve increased renin activity due to succinic acid and tissue injury due to S-adenosylmethionine and 4PY.

## Electronic supplementary material

Below is the link to the electronic supplementary material.

Supplementary Material 1

Supplementary Material 2

## Data Availability

The datasets used and/or analyzed during the current study are available from the corresponding author on reasonable request. The raw data is provided in the supplementary file. Further information that supports the findings of the metabolome analysis are available from HMT (Tsuruoka, Yamagata, Japan), but restrictions apply to the availability of these data, which were used under license for the current study, and so are not publicly available. Data are however available from the authors upon reasonable request and with permission of HMT (Tsuruoka, Yamagata, Japan).

## Abbreviations

CKD: Chronic Kidney Disease
I: Ischemic
C: Control
4PY: N1-methyl-4-pyridone-5-carboxamide
DOHaD: Developmental Origins of Health and Disease
SGA: Small-for-Gestational-Age
AMP: adenosine monophosphate
GIS: Glomerular Injury Score
TIS: Tubulointerstitial Injury Score
NAD: Nicotinamide Adenine Dinucleotide
PARP: Poly (ADP-ribose) Polymerase
