# Supplementary figures and images for: Evaluation of kidney injury and metabolomic analysis in adulthood in a non-obese hyperglycemic female mouse model after birth with low birthweight
Source: BMC Nephrol. 2025 Jul 2;26:343. doi: 10.1186/s12882-025-04290-1 (PMC12224424; doi:10.1186/s12882-025-04290-1)

Online Resource 1


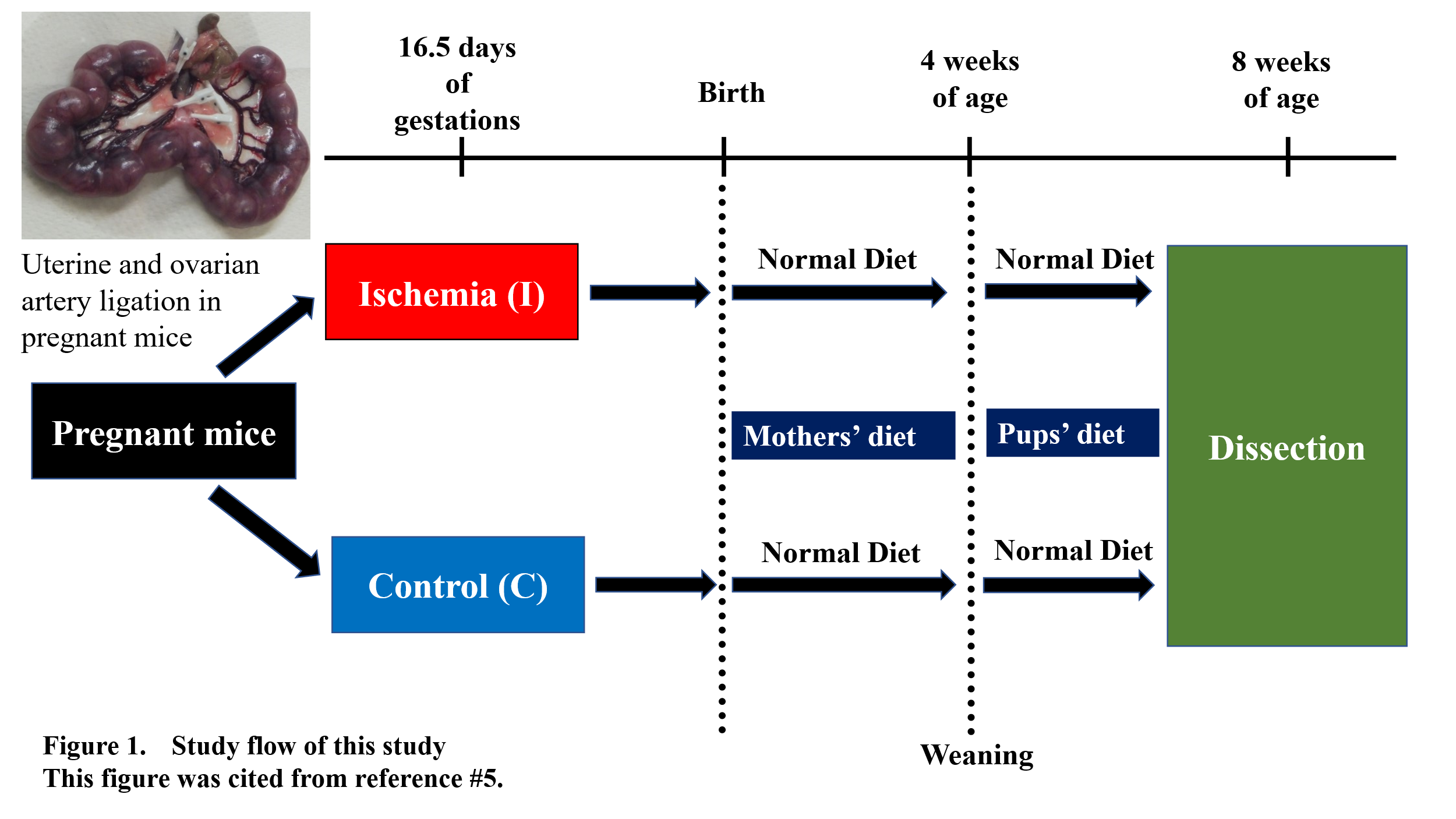

Supplement: Supplementary file 2 — Supplementary Material 2 [file 12882_2025_4290_MOESM2_ESM.docx]
